# Impact of Multidisciplinary Team Escalating Approach on Antibiotic Stewardship in the United Arab Emirates

**DOI:** 10.3390/antibiotics10111289

**Published:** 2021-10-22

**Authors:** Ahmed A. Sadeq, Jinan M. Shamseddine, Zahir Osman Eltahir Babiker, Emmanuel Fru Nsutebu, Marleine B. Moukarzel, Barbara R. Conway, Syed Shahzad Hasan, Geraldine M. Conlon-Bingham, Mamoon A. Aldeyab

**Affiliations:** 1 Department of Pharmacy, Shaikh Shakhbout Medical City in Partnership with Mayo Clinic, Abu Dhabi P.O. BOX 11001, United Arab Emirates; ahmed.sadeq@hud.ac.uk (A.A.S.); jshamseddine@ssmc.ae (J.M.S.); mmoukarzel@ssmc.ae (M.B.M.); 2 Division of Infecious Diseases, Shaikh Shakhbout Medical City in Partnership with Mayo Clinic, Abu Dhabi P.O. BOX 11001, United Arab Emirates; zahirbabiker@doctors.org.uk (Z.O.E.B.); ensutebu@ssmc.ae (E.F.N.); 3 Department of Pharmacy, School of Applied Sciences, University of Huddersfield, Huddersfield HD1 3DH, UK; b.r.conway@hud.ac.uk (B.R.C.); s.hasan@hud.ac.uk (S.S.H.); 4 Institute of Skin Integrity and Infection Prevention, University of Huddersfield, Huddersfield HD1 3DH, UK; 5 Pharmacy Department, Southern Health and Social Care Trust, Craigavon BT63 5QQ, UK; geraldine.conlonbing@southerntrust.hscni.net

**Keywords:** antimicrobial stewardship program, multidisciplinary team, days of therapy, length of stay, daily defined doses, readmission, mortality, AWaRe, patient days

## Abstract

Antimicrobial stewardship programs (ASP) are an essential strategy to combat antimicrobial resistance. This study aimed to measure the impact of an ASP multidisciplinary team (MDT) escalating intervention on improvement of clinical, microbiological, and other measured outcomes in hospitalised adult patients from medical, intensive care, and burns units. The escalating intervention reviewed the patients’ cases in the intervention group through the clinical pharmacists in the wards and escalated complex cases to ID clinical pharmacist and ID physicians when needed, while only special cases required direct infectious disease (ID) physicians review. Both non-intervention and intervention groups were each followed up for six months. The study involved a total of 3000 patients, with 1340 (45%) representing the intervention group who received a total of 5669 interventions. In the intervention group, a significant reduction in length of hospital stay (*p* < 0.01), readmission (*p* < 0.01), and mortality rates (*p* < 0.01) was observed. Antibiotic use of the WHO AWaRe Reserve group decreased in the intervention group (relative rate change = 0.88). Intravenous to oral antibiotic ratio in the medical ward decreased from 4.8 to 4.1. The presented ASP MDT intervention, utilizing an escalating approach, successfully improved several clinical and other measured outcomes, demonstrating the significant contribution of clinical pharmacists atimproving antibiotic use and informing antimicrobial stewardship.

## 1. Introduction

Antibiotics are undoubtedly among the most vital discoveries in human history, and their use has saved multitudinous lives from once-lethal infections [1]. The inappropriate use and overuse of antibiotics have played a role in the development of antimicrobial resistance [1,2,3,4]. Together with the lack of synthesis of new antibiotics, this poses significant challenges to worldwide health care, causing morbidity and increased costs of therapy and days of treatment [5,6,7,8]. Multidrug-resistant bacteria have increased hospital stays in the United States to around 8 million, with a cost of more than USD 20 billion [9].

In order to combat antimicrobial resistance and improve clinical outcomes, antimicrobial stewardship programs (ASPs) have emerged as part of the solution [10]. The appropriate implementation of ASPs in hospitals requires the involvement of a multidisciplinary team (MDT), which functions to monitor and record all aspects of antibiotic utilisation (e.g., selections and indications) with a primary focus on antibiotic use, clinical parameters, antibiotic resistance, and cost [11]. The Infectious Disease Society of America (IDSA) and the Society for Healthcare Epidemiology of America (SHEA) have disseminated guidelines to develop and enhance ASPs within healthcare organisations [12]. These guidelines recommend using specialised physicians in infectious diseases (ID) and a clinical pharmacist, with appropriate ID training, as vital ASP members and part of an ASP MDT [13].

Prospective patient case reviews of antibiotics are very crucial to improve the use of such agents, and their impact has been measured by several studies, through which the physicians themselves were the team which reviewed patient cases with antibiotic prescriptions, and the result was a modest improvement [14,15,16]. This result was in line with another study which involved MDT case reviews in a 720-bed health system and concluded that such an intervention can have a positive impact on prescribing physicians’ behaviour. Interestingly, in that study, the MDT team consisted of clinical pharmacists, an ID physician and ICU physicians, and all patients who were receiving antibiotics in the ICU were reviewed by the clinical pharmacists and were discussed for appropriate intervention [17].

In our study, we planned to use a comprehensive escalating approach. This approach allows reviewing all admitted patients who are on antibiotics, allows reviews to be more efficient, and makes better use of available resources and the involvement of the clinical pharmacists. As per our knowledge and database searching, no study to date has evaluated the impact of an ASP MDT intervention on such a cohort group of patients, or has it used a similar escalating approach. Two groups within two different periods were studied and compared, i.e., the non-intervention group (February to July 2020) and the intervention group (August to January 2021).

This study aimed to evaluate the impact of an antimicrobial stewardship program multidisciplinary team (ASP MDT) intervention on clinical, microbiological, and other relevant measured outcomes among hospitalised patients. In addition, this study aimed to highlight the clinical pharmacist’s role as a part of an ASP MDT.

## 2. Results

**Figure 1:**
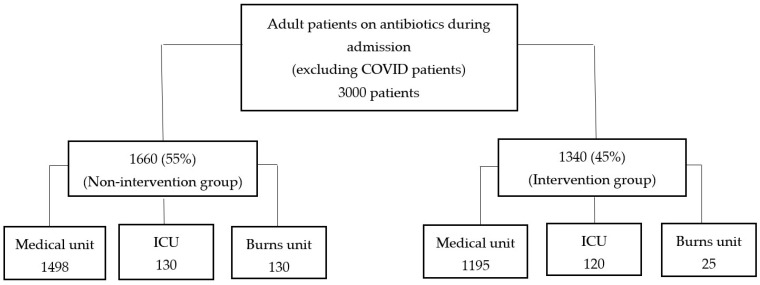
Flow chart of distribution patients included in the three clinical settings (medical, ICU, and burns unit).

The study involved a total of 3000 patients from medical, intensive care, and burns units, with 1660 (55%) patients composing the non-intervention group and 1340 (45%) the intervention group. The distribution of included patients in each group is shown in Figure 1. It should be noted that coronavirus disease (COVID-19) patients were excluded because they had different treatment plans, guidelines, and protocols (methodology section). The highest number of patients were in the medical unit for both the non-intervention group [1498 (49.9%)] and the intervention group [1340 (44%)]. The average age in the groups was 54 and 60 years, respectively. More than 50% of the patients from both periods were male, and the mean Charlson score was higher in the ASP MDT intervention period (µ = 4.75, SD = 3.621) than in the non-intervention period (µ = 3.75, SD = 3.292). Patients’ baseline characteristics and clinical outcomes are presented in Table 1.

Patients who received antibiotics were reviewed by ASP MDT (the intervention). The multidisciplinary team consisted of ID physicians, an ID clinical pharmacist, and ward clinical pharmacists. The MDT conducted an intervention that followed an escalating approach. Firstly, the ward clinical pharmacists reviewed all adult patients receiving antibiotics who were admitted in medical, intensive care, or burns units for potential ASP intervention. The ward clinical pharmacist then either intervened and discussed the recommendations with the ward physician or escalated the patient’s case to the ID clinical pharmacist who in turn reviewed the antibiotic prescription for any clinical input. If the outcome of this patient’s case review was still undecided, it was escalated to the ID physician for review and recommendations. More details about the intervention are available in the methodology section.

**Figure 2:**
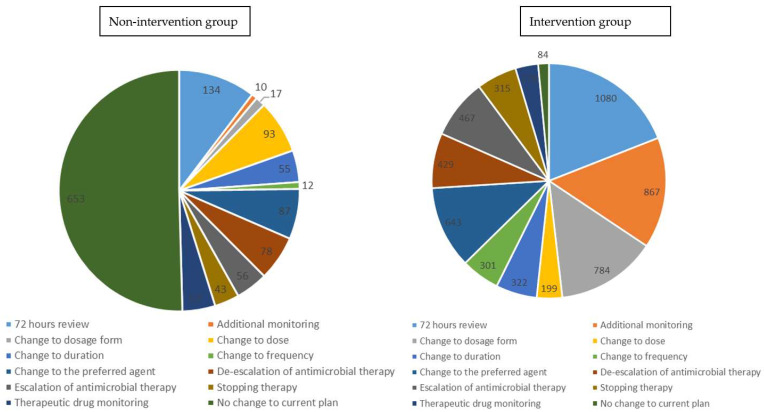
ASP recommendations for the non-intervention and intervention groups.

During the study period, twelve different types of interventions were implemented and documented in the patient’s electronic records, with ‘72 h review’ being the most used intervention and ‘therapeutic drug monitoring’ being the least (Figure 2). A total of 5669 interventions were performed during the intervention period while only 653 interventions were undertaken in the non-ASP MDT intervention period.

The unadjusted regression (not adjusted for age, gender, and Charlson score) analysis of the clinical outcomes for different ward settings showed medical wards had significant differences (*p* < 0.01) for all clinical outcomes except for the DOT (*p* = 0.201), as shown in Table S1.

The results of multiple regression (adjusted for age, gender, and Charlson score) analysis, comparing the non-intervention group with the intervention group in the medical setting, is shown in Table 2. The following outcomes, adjusted for age, gender, and Charlson score were shown to significantly improve in the intervention period: LOS (coefficient = −0.25, *p* < 0.01), readmission (*p* < 0.01; OR = 0.67; 95% CI = 0.55, 0.80), and mortality (*p* < 0.01; OR = 0.58; 95% CI = 0.43, 0.78). Although days of antibiotic therapy were decreased, the reduction was statistically insignificant (coefficient = −1.17, *p* = 0.243).

The use of IV antibiotics decreased in the ASP MDT intervention period compared with the non-intervention period.

In the medical wards during the non-intervention period, there were a total of 6414 IV orders and 1333 oral orders, while during the intervention period, there were 4394 IV orders and 1079 oral orders. The ratio of IV-to-oral antibiotics in medical wards decreased from 4.8 in the non-intervention period to 4.1 in the intervention period.

Regarding surgical patients in the study site, the intervention was not implemented in both periods of the study. Analysis of surgical patient data showed that readmissions (*p* = 0.592; OR = 1.06; CI = 0.84, 1.35) and mortality (*p* = 0.845; OR = 0.92; CI = 0.40, 2.09) were not changed significantly between non-intervention and ASP MDT intervention periods (Table S2). Conversely, LOS (*p* < 0.01; mean difference = −0.22) and DOT (*p* < 0.01; mean difference = −0.28), increased significantly during the intervention period.

Consumption, based on total Defined Daily Dose adjusted per 100 patient days (DDD/100 PD), showed that the highest percentage was for combinations of penicillins including β-lactamase inhibitors in both non- and ASP MDT intervention periods (56.53 and 43.54, respectively). The lowest use percentage was for second-generation cephalosporins and polymyxins in the non-intervention period (0.26%) and nitrofuran derivatives in the intervention period (0.001%). The lincosamides class achieved the highest increase in DDD/100 PD (in %) amongst all other classes in the ASP MDT intervention period compared to the non-intervention period (Relative Rate Change [RRC = 3.52]), whilst first-generation cephalosporins achieved the highest reduction (RRC = 0.50) (Table S3).

In the medical setting, DDD/100 PD (in %) of antibiotics with Access category decreased in the ASP MDT intervention period (RRC = 0.79), while Watch and Reserve categories increased (RRC = 1.09 and 1.19, respectively; Table 3). Combining the three settings (i.e., medical, ICU, and burns) showed a decrease in both Access (RRC = 0.76) and Reserve (0.66) categories while there was an increase in Watch.

Fourteen antibiotics accounted for 90% of total antibiotic use in the non-intervention period, with five Access antibiotics, eight Watch, and one antibiotic from the Reserve category. On the other hand, thirteen comprised 90% of total antibiotic use in the intervention period, with the same numbers for two AWaRe (Access, Watch and Reserve) categories as in the non-intervention period and four antibiotics from the Access category. Piperacillin/tazobactam had the highest percentage of DDD/100 PD in both periods as it increased from 27% in the non-intervention period to 38% in the intervention period (Table 4).

Concerning microbiological outcomes, there were no cases of *Clostridium difficle* (C.diff) during the non-intervention period and only six cases during the ASP MDT intervention period. Thirty-day all-cause readmission rates for patients with pneumonia or urinary tract infection (UTI) were 0.13 and 0.2, respectively, in the non-intervention period, and 0.16 and 0.2 in the intervention group. Regarding methicillin-resistant *Staphylococcus aureus* (MRSA) and multidrug-resistant organisms (MDRO) bloodstream infections, a total of 23 cases during the non-intervention period and 14 cases during the ASP MDT intervention period were reported (Table S4).

The number of extended spectrum B-Lactamase (ESBL) producing bacteria cultures in the non-intervention group was 767 (community acquired: 528; hospital acquired: 239), while in the intervention group the number was 891 (community acquired: 698; hospital acquired: 193) as shown in Table S5.

The total cost saving from antibiotics was AED 11,119 and from LOS was AED 6,013,650. Combining both values, the total cost saving was AED 6,024,769, equivalent to USD 1,637,165 (Table 5).

## 3. Discussion

Our study demonstrated a comprehensive approach to improve and optimise antibiotic use in hospitals. This approach used a multidisciplinary team of ID physicians, ID clinical pharmacist, and ward clinical pharmacists. The multidisciplinary team, which represents the hospital antimicrobial stewardship program, used an escalating approach by reviewing all adult patients admitted to medical, intensive care, and burns units who are on antibiotic therapy. The escalating approach started with ward clinical pharmacists, who reviewed all the patients receiving antibiotics in their respective wards. For the cases where the ward clinical pharmacist could not decide (complex cases), they escalated these cases to the ID clinical pharmacist, who made decisions in some of those referred cases. Others were escalated to the ID physicians for the final decision. It should also be noted that some complex cases were directly reviewed by the ID physician to make a final decision on the antibiotic therapy plan. During case review, the ASP MDT followed the hospital antimicrobial guidelines to ensure that the patients received the best possible antibiotic therapy.

LOS decreased significantly in the ASP MDT intervention period compared to the non-intervention period when adjusted by age, gender, and Charlson scores. This reduction might have been driven by the ‘IV-to-oral antibiotic switch’ intervention, which allowed patients to complete their course of antibiotics at home instead of staying at the hospital. Improving the appropriateness of antibiotic prescribing resulted in faster and more effective recovery from the active infection. The following interventions achieved this: changing to preferred agent, escalation, de-escalation, changing duration, 72 h review, and therapeutic drug monitoring. In one meta-analysis, fifteen randomised control trials (RCTs) were analysed for the effect of successful ASP MDT intervention on decreasing length of hospital stay. It concluded there was a mean reduction of 1.12 days in the intervention group [17]. In another study, the length of hospital stay fell by an average of one day in patients admitted to a surgical ward who received an intervention that consisted of educating physicians, handing out pocket-sized cards, and providing IV-to-oral switch advice in the electronic patient record [18].

Reduction in days of antibiotic therapy was attempted in our study by strictly applying the hospital antimicrobial guidelines in terms of duration of antibiotic therapy and the criteria to whether to continue or discontinue antibiotic treatment and IV-to-oral antibiotic switch. Although there was a reduction in DOT in the medical setting, the reduction was not statistically significant. The most likely reason for this is ID physicians’ encouragement to the attending primary teams to use shorter durations of antibiotic courses during the non-intervention period for the admitted patients, in accordance with hospital antimicrobial guidelines. One of the criteria mentioned in the hospital guidelines to determine the duration of therapy is the trend of procalcitonin. This method has accomplished a reduction in days of antibiotic therapy and a decrease in daily defined antibiotic doses in critically ill patients in an open-labelled randomised controlled trial [19]. In another study, ASP MDT interventions led by a surgeon, and included a pharmacologist and an ID specialist, decreased the duration of carbapenem therapy in general surgery services by providing recommendations on appropriate antibiotic therapy [20]. A C-reactive protein (CRP)-based protocol for antibiotic therapy was compared with a control group in critical care settings, and it did not result in a statistically significant difference in the duration of antibiotic treatment days between both groups [21].

One of the measures that indicates the effectiveness of antibiotic therapy that patients received is the readmission rate within 30 days due to any cause (all-cause readmission including infections), and in-hospital mortality. Our study showed that these outcomes decreased significantly in the intervention group in the medical setting. One of the reasons that a patient is re-admitted to the hospital within thirty days is infection relapse. By choosing the appropriate antibiotic, the chance of targeting the right bacteria at the right site of infection by the right antibiotic increases. This can increase the infection cure rate and decrease the chance of infection relapse, therefore decreasing readmission and mortality rates [22,23,24]. All-cause 30-day-readmissions for patients with urinary tract infection or pneumonia and rate of MDRO and MRSA bloodstream infections were few in this study. A prospective observational study of carbapenem prescriptions measured de-escalation performance and 30-day readmission rates in pneumonia patients through an ASP intervention involving a pharmacist. However, it did not decrease the readmission rate [25]. Readmission for UTI patients was assessed in a retrospective chart review comparing outcomes between two different periods, and this intervention proved to be effective in reducing readmission rates [26]. Staphylococcal bloodstream infection was investigated in one particular study through conducting unsolicited standardised form-based ASP and resulted in a reduction in the 30-day readmission rate. However, this was not statistically significant [27]. In the same study, the mortality rate was also measured, and there was again no significant difference when compared with the control group. All intervention types used in our study contributed to effective antibiotic therapy courses.

Our intervention successfully decreased the IV-to-oral antibiotic ratio. The criteria for switching were outlined in the hospital antimicrobial guidelines and were followed by the ASP MDT. Effective IV-to-oral switches have been reported elsewhere [28,29,30]. Moreover, IV-to-oral switching has always been considered a significant factor in reducing the length of hospital stay, negating the requirement to remain in hospital to receive IV antibiotics. A history-controlled intervention study investigated this relationship between IV-to-oral switching and length of hospital stay, and found a statistically significant decrease in hospital stay [28]. However, in three other studies, the length of hospital stay was not reduced significantly [15,28,30]. Although the relationship between these two parameters (IV-to-oral and length of hospital stay) could not be accurately measured in our study due to other factors influencing patient hospital stay, the safety of switching to an oral antibiotic was determined by measuring readmission and mortality rates within 30 days, and as mentioned above, these were found to decrease.

Cost-saving was mainly due to a reduction in the days of hospital stay. Medication cost was not reduced markedly owing to the increased use of some high-cost broad-spectrum antibiotics in the intervention group (e.g., ceftazidime/avibactam). These antibiotics were appropriate for some infections caused by bacteria that were sensitive to those antibiotics in the microbial cultures, and that was justified by the increase in the number of cultures growing ESBL-producing bacteria in the intervention group. This result was in line with other studies. A trial of interventions by an Antimicrobial Management Program team reported a reduction in therapy cost, but the reduction was not statistically significant [31]. In a further study, clinical pharmacist interventions in an infectious disease ward did not significantly decrease direct medication cost for the patients [32]. However, savings of more than USD 900 were achieved on antibiotics in a study involving an ID physician, a clinical microbiologist with experience in pharmacokinetics and pharmacodynamics, a laboratory microbiologist, two pharmacists, an internal medicine specialist, and a computerised system analyst [33].

Microbiological outcomes in our study included the rate of MDRO and the rate of MRSA bloodstream infections. Many articles have investigated different microbiological outcomes, including outcomes that are similar to ours. Yamada et al. conducted a study to determine the impact of the intervention of an ASP team, including an ID physician and pharmacist, on several outcomes, including the rate of Gram-negative multidrug-resistant bacteria (Citrobacter, Enterobacter, and Acinetobacter species). It concluded that the effect of intervention provided by the team successfully decreased the resistance rates of the studied bacteria [34]. On the other hand, a prospective interrupted time-series study performed in one hospital in the USA between 2003 and 2007 evaluated the effect of ASP intervention on the rates of multidrug-resistant bacteria, and found that there were no reduction in resistance rates of *Pseudomonas aeruginosa, Enterobacter cloacae, E.coli*, or *Klebsiella pneumoniae* [35]. Similar findings were reported from a study conducted exclusively in an ICU setting [36]. Another microbiological outcome that we assessed for this study was the number of cultures growing ESBL-producing bacteria. The results showed that there was an increase in the number of this bacteria (community-acquired ESBL-producing bacteria) in the intervention group, which requires the use of more broad-spectrum antibiotics. Of interest, the number of hospital-acquired ESBL-producing bacteria decreased during the intervention group. Overall, our study showed that the evaluated clinical outcomes were improved, highlighting the appropriateness of antibiotic choice which was ensured by the ASP MDT.

There were six hospital-acquired C.diff cases in the intervention period compared to zero cases during the non-intervention period. This warrant further investigations by the hospital’s Infection Prevention and Control (IPC) team. Defined daily dose (DDD) was measured for each antibiotic, WHO class, and AWaRe category and then adjusted per 100 patient days. The highest increase in antibiotic class was for combinations of penicillins. It increased during the intervention period despite the reduction in co-amoxiclav DDD/100 PD%. Piperacillin/tazobactam mainly drove this increase as its use is recommended in the hospital local guidelines. The increase in piperacillin/tazobactam use led to increased use of the Watch category during the intervention period, while the reduction in co-amoxiclav DDD/100 PD% resulted in decreased use of the Access category.

The findings of this study highlighted the role of the clinical pharmacist as an active member in the ASP MDT, participating effectively in improving antibiotic use. In a study conducted in Europe, which compared antibiotic therapy decisions taken by ID physicians alone with the decisions taken by a team of ID physicians and a hospital pharmacist to improve clinical and economic outcomes, findings favoured having a physician and pharmacist team [37]. Many other studies have achieved positive outcomes by involving pharmacists as a part of an ASP MDT [31,32,33].

Our approach allowed the review of all patients admitted into the wards who were on antibiotics instead of selecting specific cases. The relative availability of clinical pharmacists enabled this approach. Previous studies have been conducted on a specific group of patients, for example, those who are only on IV antibiotics, surgical patients, critical care setting patients, infectious disease ward, or specific cases of certain infections [15,20,21,31]. Therefore, the advantages of our approach encompass including a wider number of patients to benefit from the intervention offered by the ASP MDT, and this improved clinical outcomes and other measures such as therapy cost and IV-to-oral switching in the medical, intensive care, and burns units within a six-month period.

This study has some limitations. Firstly, there was a period of coronavirus disease (COVID-19) surge during the study, from April until June 2020. However, the study site hospital was not designated for COVID-19 patients only and continued to operate for other medical services. During that period, the ASP team launched a campaign (increasing awareness of antibiotic use in COVID-19 patients through emails) to control the inappropriate use of antibiotics for those patients; in order to reduce the impact of the COVID-19 surge, all COVID-19 patients were excluded from the study. Secondly, there were some interventions and reviews performed by the ID team during the non-intervention period. However, these interventions were conducted on specific patient cases, which comprised less than 20% of total patients on antibiotics. In addition, ASP interventions performed by the ID physicians specifically were not able to be determined electronically since the intervention checklist tool was accessible to clinical pharmacists only. Nevertheless, ID physician interventions and plans were agreed with clinical pharmacists who in turn documented them using the ASP intervention electronic tool. Furthermore, types and severity of infectious diseases were not able to be accurately obtained from patients’ electronic health records. It was not possible to control for potential variation in factors, such as social behaviours, climate, food, and water consumption during both study periods. However, we observed no concerning changes of the assessed clinical outcomes for surgical patients who were not exposed to the intervention during both study periods. Finally, there was no randomisation in our study; nevertheless, from an ethical point of view, we decided to apply the ASP MDT intervention on all patients who received antibiotics to ensure all adult patients in the three settings were getting the benefit of the intervention.

## 4. Materials and Methods

### 4.1. Clinical Settings

This study was conducted in Shaikh Shakhbout Medical City (SSMC), a 741-bed governmental tertiary hospital in Abu Dhabi/UAE. This hospital provides medical, surgical, and ICU facilities and serves a population of 2.7 million inhabitants.

### 4.2. Study Design

We conducted a quasi-experimental study (before–after) involving two groups of patients in two different periods over a 12-month time frame. For the first six months (non-intervention period; February to July 2020), patients admitted to the medical, intensive care, and burns units and who were prescribed antibiotics at any point of time during admission were retrospectively reviewed. Over the following six months (intervention period; August 2020 to January 2021), patients, with the same criteria as above, who were prospectively reviewed and received the ASP MDT intervention. Outcomes were compared between the non-intervention (reference) and the intervention groups.

Patient cohorts were divided into three subgroups: medical unit, ICU, and burns unit. Each subgroup from the non-intervention group was compared with its parallel in the intervention group, and patients from three settings combined were compared as non-intervention group versus intervention group.

Patients admitted to surgical wards and who received antibiotics were included as a control group to validate the study findings. These patients did not receive the ASP MDT intervention for the whole 12-month period. They were divided as per the time frame of the aforementioned periods to assess whether there were any changes in the clinical outcomes without receiving the intervention.

### 4.3. Inclusion and Exclusion Criteria

Adult patients hospitalised in medical, intensive care, and burns units for any morbidity and who were prescribed an antibiotic to treat or to prevent an infection during their hospital stay were included. Patients aged under 18 years, hospitalised patients without an antibiotic prescription, and COVID-19 patients were excluded from the study. COVID-19 patients were excluded because they had different treatment plans, guidelines, and protocols. In addition, their clinical outcomes might be affected markedly due to other comorbidities as a result of COVID-19.

### 4.4. Intervention

Patients who received antibiotics were reviewed by the ASP MDT (the intervention). The multidisciplinary team consisted of ID physicians, an ID clinical pharmacist, and ward clinical pharmacists. The MDT conducted an intervention that followed an escalating approach. Firstly, the ward clinical pharmacists reviewed all adult patients receiving antibiotics who were admitted in medical, intensive care, or burns units for potential ASP intervention. The ward clinical pharmacist then either intervened and discussed the intervention with the ward physician or escalated the patient’s case to the ID clinical pharmacist if they could not make a decision due to the complexity of the case, or if there was not enough information in terms of cultures and sensitivity results. After receiving the case from the ward clinical pharmacist, an ID clinical pharmacist reviewed the antibiotic prescription for any clinical input. If the outcome of this patient’s case review was still undecided (reasons include complex cases, cases that require physician intervention, or when there is shared decisions need to be taken), it was escalated to the ID physician for review and recommendations. For some complicated cases (e.g., bacteraemia, endocarditis, or CNS infections) the ID physicians directly reviewed and provided their recommendations. It should be noted that the hospital antimicrobial guidelines, which were based on the local antibiogram and several international guidelines and UpToDate^®^ E-library, informed the interventions implemented as part of the ASP MDT.

A standard checklist tool of ASP MDT recommendations which represent the ASP intervention was created in the electronic patients’ profiles (using Cerner^®^ Hospital Health System) and recommendations uploaded in this tool were prepared and agreed upon by a group of healthcare experts comprising senior clinical pharmacists and consultant physicians. Using this ASP intervention electronic tool, the clinical pharmacists documented the interventions that had been performed. Intervention types (recommendations) included in the checklist were 72-h review (to follow for culture and sensitivity), antibiotic change, escalation, de-escalation, discontinuing therapy, dose change, duration change, frequency change, dosage form change, therapeutic drug monitoring, or no change to current care.

### 4.5. Data Collections and Outcomes

The following data were obtained from the hospital Cerner^©^ healthcare system electronic records: age, gender, length of hospital stay, days of antibiotic treatment, readmission within 30 days, all-cause 30-day readmission rate for patients with pneumonia, all-cause 30-day readmission rate for patients with Urinary Tract Infection (UTI), in-hospital mortality rate, route of antibiotic administration, antibiotics consumption, and cost. In addition, microbiological data, including the number of cases of bloodstream infections caused by *Methicillin-resistant staphylococcus aureus* (MRSA) and multidrug-resistant organisms (MDRO), number of cultures growing ESBL-producing bacteria, and adult hospital patient days, were obtained.

MDRO include MRSA, extended-spectrum B-lactamase (ESBL), Escherichia *coli* (E. coli), *Klebsiella pneumonia* (K. pneumonia), *Vancomycin-resistant Enterococci* (VRE), *Acinetobacter baumanii*, *Vancomycin-resistant Staphylococcus-aureus*, and other organisms that are resistant to most available antimicrobial agents.

The pre-intervention and intervention groups were compared using the following clinical outcomes: length of hospital stay, days of antibiotic treatment, readmission for any infectious disease within 30 days, all-cause readmission rate for patients with pneumonia within 30 days, all-cause readmission rate for patients with UTI within 30 days, in-hospital mortality rate, IV-to-oral antibiotics, therapy cost, and MRSA-and MDRO- bloodstream infections adjusted per 100 patient days.

Length of hospital stay (LOS) was calculated as the difference between admission and discharge dates. Days of antibiotic therapy (DOT) were calculated as the total number of days the patient received an antibiotic. IV-to-oral antibiotics were measured by dividing the number of IV antibiotic orders (numerator) by the number of oral antibiotic orders (denominator). Readmissions within 30 days and mortality during the hospital stay were indicated for each patient as ’yes’ or ‘no’ for the first and ’deceased’ or ‘not deceased’ for the latter. To calculate percentages, the number of ‘yes’ for the readmission and the number of ‘deceased’ for the mortality was divided by the total number of patients in the corresponding group (non-intervention or intervention group) in each setting.

Microbiological outcomes, including the rate of MDRO- bloodstream infections and MRSA- bloodstream infections per 100 patient days (PD), and Clostridioides *difficile*, were calculated by dividing the number of cases over the adult patients days for all three settings combined, and the product was multiplied by 100. Number of cultures growing ESBL producing bacteria has also been identified as a microbiological outcome.

The assigned DDD by the WHO/Anatomical Therapeutic Chemical (ATC) index for each antibiotic for systemic use (J01) was employed and was expressed as DDD per 100 patient days [38] To compare antibiotic DDDs/100 PD between the non-intervention and intervention periods, relative rate change (RRC) was measured by dividing the percentage of DDD/100 PD (DDD/100 PD%) for each antibiotic within the intervention by the same within the non-intervention period. Antibiotics were classified as per WHO/ATC classification criteria and categorised as per the WHO AWaRe (Access/Watch/Reserve) categorisation [38,39]

Drug Utilization 90% (DU90%) is an indicator used to highlight the most frequently used antibiotics by identifying agents that account for 90% of DDD/100 PD% [39]. Antibiotics were ordered in descending manners in terms of DDD/100 PD (in %), and antibiotics constituting 90% were determined.

The non-intervention and intervention groups’ baseline characteristics (age, gender, and comorbidities) were described and compared. Comorbidities were assessed using the Charlson comorbidity index (CCI) [40,41]

Cost saving was determined as follows: the difference in antibiotics consumption was multiplied by the purchasing cost, and the cost of the difference in-hospital stay in days was calculated by multiplying the standard cost for hospital patient day for each setting (medical, intensive care or burns units) by the difference in days of hospital stay. The cost is presented in Arab Emirates Dirham (AED).

### 4.6. Statistical Analysis

Patients’ baseline characteristics and clinical outcomes were described using means and standard deviations for continuous data while frequency and percentages for categorical data, and were compared using independent sample t-test and chi-squared test, respectively. Logistic and/or linear regression analysis presented unadjusted estimates for four clinical outcomes (LOS, DOT, 30-day readmission, and in-hospital mortality) of all ward settings combined and separately. Outcomes with *p*-values less than 0.25 were chosen for the second phase (reference: non-intervention group), which comprised backward multiple regression analysis adjusted for age, gender, and Charlson score. In order to meet the independent t-test and regression assumption for normality of the continuous variables (particularly LOS and DOT), a natural logarithm was calculated to represent the original variables. In the final model, *p*-values below 0.05 were considered statistically significant.

Frequency of intervention types (72-h review, antibiotic change, escalation, de-escalation, discontinuing therapy, dose change, duration change, frequency change, dosage form change, therapeutic drug monitoring, or no change to current plan) applied to both groups of patient and microbiological outcomes were presented using descriptive analysis.

As patients in the surgical department did not receive any intervention over both periods, their outcomes were compared using only independent sample *t*-test and Chi-squared test.

## 5. Conclusions

An antimicrobial stewardship program multidisciplinary team escalating intervention successfully improved patients’ clinical outcomes in the medical wards in terms of length of hospital stay, readmission, and mortality, but did not significantly impact days of antibiotic therapy or clinical outcomes in the intensive care and burns units. In addition, the intervention was also able to decrease daily defined doses adjusted per 100 patient days for antibiotics that belong to reserve WHO AWaRe categorization. Other improved outcomes include the IV-to-oral antibiotic ratio and therapy cost. The role of clinical pharmacists played a big part in the success of the ASP intervention. This highlighted the impact and the influence that the clinical pharmacist can deploy in the stewardship era. More efforts are needed to investigate ASP MDT interventions to improve clinical outcomes in intensive care and burns units.

## Figures and Tables

**Table 1:** Patients’ baseline characteristics and clinical outcomes comparison between non-intervention and ASP MDT intervention groups.

Setting-	Medical			ICU			Burns			Total		
	Non-Intervention *n* = 1498	Intervention *n* = 1195	*p*	Non-Intervention *n* = 130	Intervention *n* = 120	*p*	Non-Intervention *n* = 32	Intervention *n* = 25	*p*	Non-Intervention *n* = 1660	Intervention *n* = 1340	*p*
Mean age (years)	54 (±18.472)	60 (±20.637)	<0.01	59 (±18.734)	62 (±22.973)	0.280	36 (±12.569)	42 (±19.269)	0.559	54 (±18.632)	60 (±21.002)	<0.01
Gender			<0.01			0.053			0.599			<0.01
Male	1063 (71)	693 (58)		93 (72)	77 (64)		24 (75)	17 (68)		1185 (71)	787 (59)	
Female	435 (29)	502 (42)		32 (28)	43 (36)		8 (25)	8 (32)		475 (29)	553 (41)	
Charlson score	3.75 (±3.292)	4.75 (±3.621)	<0.01	6.2 (±3.639)	6.9 (±4.438)	0.171	1.3 (±1.481)	2 (±2.372)	0.135	3.9 (3.382)	4.9 (3.751)	<0.01
Mean LOS	12 (±15.999)	9.5 (±14.750)	<0.01	21.3 (±22.839)	17 (±16.240)	0.124	30.6 (±19.510)	25.9 (±22.444)	0.318	13 (±17.273)	10.5 (±15.090)	<0.01
Mean DOT	16.049 (±26.555)	15.1 (±21.904)	<0.01	43.429 (±57.977)	46.650 (±90.300)	<0.01	27.7 (±31.950)	44.41 (±86.536)	<0.01	18.3 (±31.137)	18.3 (±36.137)	<0.01
Readmission rate Yes No	27% 397 (27) 1101 (73)	20% 237 (20) 958 (80)	<0.01	5% 7 (5) 118 (95)	5% 6 (5) 114 (95)	0.665	0 32 (100) 0	0 32 (100) 0	NA	24% 403 (24) 1257 (76)	28% 244 (18) 1096 (82)	<0.01
Mortality rate Deceased Not deceased	13% 189 (13) 1309 (87)	9% 104 (9) 1091 (91)	<0.01	68% 89 (68) 36 (32)	66% 79 (66) 41 (34)	0.331	9% 3 (9) 29 (91)	4% 1 (4) 24 (96)	0.431	17% 285 (17) 1375 (83)	16% 184 (14) 1556 (86)	0.01

*n* represents the number of patients in the group; data presented as *n* (%); data presented as mean ± SD; NA: no cases found; LOS: length of hospital stay in days; DOT: days of antibiotic therapy.

**Table 2:** Multiple regression analysis comparing non-intervention with ASP MDT intervention groups in a medical setting.

Terms-	LOS		DOT		Readmission		Mortality	
	*p*	Coefficient (95% CI)	*p*	Coefficient (95% CI)	*p*	OR (95% CI)	*p*	OR (95% CI)
Groups	<0.01	−0.25 (−0.33, −0.18)	0.243	−1.167 (−3.12, 0.79)	<0.01	0.669 (0.55, 0.80)	<0.01	0.58 (0.43, 0.78)
Gender	<0.01	−0.10 (−0.18, −0.02)	0.886	0.874 (−1.17, 2.91)	0.01	0.773 (0.635, 0.940)	<0.01	1.54 (1.14, 2.06)
Age	<0.01	0.008 (0.004, 0.011)	0.401	−0.006 (−0.09, 0.07)	0.025	1.009 (1.001, 1.017)	<0.01	1.03 (1.02 1.05)
Charlson score	0.311	0.006 (−0.014, 0.027)	0.055	0.459 (−0.009, 0.92)	0.897	0.997 (0.954, 1.043)	<0.750	1.01 (0.94, 1.08)

Regression was adjusted for gender, age and Charlson score. LOS: Length of hospital stay in days; DOT: Days of therapy; OR: odds ratio.

**Table 3:** Percentage of total antibacterial consumption expressed as DDD/PD categorised by WHO AWaRe category for the non-intervention and ASP MDT intervention periods.

	Non-Intervention Period		Intervention Group Period		
AWaRe Category	DDD/100 PD	%	DDD/100 PD	%	RR Change
Medical					
Access	21.10	29%	18.42	23%	0.79
Watch	48	67%	58.55	72%	1.09
Reserve	3.24	4%	3.86	5%	1.19
ICU					
Access	8.90	13%	13.10	13%	0.96
Watch	44.50	66%	80.10	78%	1.17
Reserve	13.53	21%	9.40	9%	0.45
All settings					
Access	12.00	29%	9.60	22%	0.76
Watch	27.10	65%	32.60	74%	1.14
Reserve	2.9	7%	2	5%	0.66

RR change: relative rate change, which is calculated as DDD per 100 PD% in the intervention period/DDD per 100 PD in the non-intervention period; PD: patient days; All settings: medical, intensive care, and burns units; AWaRe: Access, Watch and Reserve.

**Table 4:** Antibacterial consumption in all three settings (medical, ICU, burns), contributing to 90% of total antibiotic use in the non-intervention and ASP MDT intervention periods, and categorised by WHO AWaRe category.

Non-Intervention Period			Intervention Period		
Antibiotic	DDD/100 PD	DDD/100 PD%	Antibiotic	DDD/100 PD	DDD/100 PD%
Piperacillin/tazobactam	11.4	27%	Piperacillin/tazobactam	16.8	38%
Amoxicillin/clavulanate	7.1	17%	Meropenem	4.1	9%
Meropenem	3.7	9%	Flucloxacillin	3.4	8%
Azithromycin	2.6	6%	Ceftriaxone	3.1	7%
Vancomycin	2.1	5%	Amoxicillin/clavulanate	2.7	6%
Cefepime	2.1	5%	Vancomycin	2.2	5%
Linezolid	2.0	5%	Cefepime	1.8	4%
Ertapenem	1.6	4%	Linezolid	1.3	3%
Flucloxacillin	1.6	4%	Ertapenem	1.1	2%
Ceftriaxone	1.0	2%	Ceftazidime	1.1	2%
Co-trimoxazole	0.8	2%	Amikacin	1.0	2%
Amikacin	0.6	1%	Azithromycin	0.9	2%
Ampicillin	0.5	1%	Co-trimoxazole	0.6	1%
Ceftazidime	0.5	1%			

DDD: defined daily dose. PD: patient day. AWaRe: Access/Watch/Reserve; Color code: Green = Access, amber = Watch, Red = Reserve.

**Table 5:** Cost saving analysis (AED) comparing the non-intervention group with the intervention period.

Terms	Non-Intervention Period	Intervention Period	Cost-Saving
Antibiotics costs	1,644,913	1,633,794	11,119
Days of length of stay cost (Days multiplied by standard room cost)	Medical: 17,950 × 750 = 13,462,500	Medical: 11,535 × 750= 8,651,250	6,013,650
	ICU: 2719 × 1200 = 3,262,800	ICU: 2049 × 1200 = 2,458,800	
	Burns: 980 × 1200 = 1,176,000	Burns: 648 × 1200 = 777,600	
	Total = 17,901,300	Total = 11,887,650	
Total cost saving	6,013,650 + 11,119 = 6,024,769 AED		

AED: Arab Emirates Dirham. 1 USD = 3.68 AED (on 12 August 2021).

## Data Availability

Data were analysed and presented in this paper.

## Supplementary Materials

The following are available online at https://www.mdpi.com/article/10.3390/antibiotics10111289/s1. Table S1: Unadjusted regression analysis of the clinical outcomes for different ward settings comparing non-intervention and ASP MDT intervention groups. Table S2: Clinical outcomes comparison between non-intervention and ASP MDT intervention periods in a surgical setting. Table S3: Antibacterial consumption expressed in DDD/100 patient day per antibiotic class for non-intervention and ASP MDT intervention periods. Table S4: Reported microbiological outcomes for the non-intervention and ASP MDT intervention periods. Table S5: Number of cultures growing ESBL producing bacteria
