# Supplementary material for: Impact of Multidisciplinary Team Escalating Approach on Antibiotic Stewardship in the United Arab Emirates
Source: Antibiotics (Basel). 2021 Oct 22;10(11):1289. doi: 10.3390/antibiotics10111289 (PMC8614643; doi:10.3390/antibiotics10111289)
Supplement: Supplementary file 1 [file antibiotics-10-01289-s001.zip › antibiotics-1401409-supplementary.pdf]

**Table S1: Unadjusted regression analysis of the clinical outcomes for different ward settings comparing non-intervention and ASP MDT intervention groups.**

| | LOS | | DOT | | Readmission | | Mortality | |
|---|---|---|---|---|---|---|---|---|
| | p | Coefficient (95% CI) | p | Coefficient (95% CI) | p | OR (95% CI) | p | OR (95% CI) |
| **Medical** | | | | | | | | |
| | < 0.01 | -0.21 (-0.29, -0.14) | 0.201 | -1.187 (-3.007, 0.634) | < 0.01 | 0.66 (0.54, 0.79) | 0.120 | 0.79 (0.59, 1.06) |
| **ICU** | | | | | | | | |
| | 0.15 | 2.42 (2.27, 2.56) | 0.770 | 2.71 (-15.55, 20.97) | 0.665 | 1.28 (0.41, 3.92) | 0.331 | 0.76 (0.44, 1.31) |
| **Burns** | | | | | | | | |
| | 0.31 | -0.18 -0.53, 0.17) | 0.380 | 13.63 (-17.27, 44.54) | NA | NA | 0.444 | 2.48 (0.03, 4.12) |
| **Combined** | | | | | | | | |
| | <0.01 | -.22 (-0.30, -0.15) | 0.830 | -0.26 (-2.63, 2.11) | < 0.01 | 0.69 (0.58, 0.83) | 0.010 | 0.76 (0.62, 0.93) |

SD: Standard deviation; OR: Odds Ratio; LOS: Length of hospital stay in days; DOT: Days of therapy; NA: No cases found; Charlson score: comorbidity index score; Group: Non-intervention vs ASP MDT intervention group.

The non-intervention group was taken as the reference group.

**Table S2: Clinical outcomes comparison between non-intervention and ASP MDT intervention periods in a surgical setting**

| | LOS | | | DOT | | | Readmission | | | | Mortality | | | |
|---|---|---|---|---|---|---|---|---|---|---|---|---|---|---|
| | Mean | p | MD (95% CI) | Mean | p | MD (95% CI) | Yes n | No n | p | OR (95% CI) | Yes n | No n | p | OR (95% CI) |
| Periods | | < 0.01 | - 0.22 (- 0.30, - 0.14) | | < 0.01 | - 0.28 (-0.39, - 0.17) | | | 0.592 | 1.06 (0.84, 1.35) | | | 0.845 | 0.92 (0.40, 2.09) |
| Non-intervention | 9.25 | | | 1.47 | | | 133 | 1312 | | | 11 | 1434 | | |
| ASP MDT Intervention | 9.28 | | | 1.75 | | | 167 | 1543 | | | 12 | 1698 | | |

LOS: length of hospital stay; DOT: days of antibiotic therapy; OR: odds ratio; MD: mean difference using the natural logarithm; n: number of cases

**Table S3: Antibacterial consumption expressed in DDD/ 100 patient day per antibiotic class for non-intervention and ASP MDT intervention periods**

| | Non-intervention period | | | ASP MDT Intervention period | | | |
|---|---|---|---|---|---|---|---|
| Antibiotic Class | Oral DDD/100 PD | DDD/100 PD | Class % | Oral DDD/100 PD | DDD/100 PD | Class % | RR change |
| Other aminoglycosides | 0 | 0.8 | 1.03% | 0 | 0.6 | 0.78% | 0.75 |
| Penicillins with extended-spectrum | 0.03 | 1.1 | 1.41% | 0.3 | 1 | 1.29% | 0.91 |
| Macrolides | 1.6 | 6.1 | 7.84% | 1.7 | 3.8 | 4.91% | 0.63 |
| First-generation cephalosporins | 0 | 0.4 | 0.51% | 0 | 0.2 | 0.26% | 0.50 |
| Fourth-generation cephalosporins | 0 | 3.3 | 4.24% | 0 | 1.9 | 2.45% | 0.58 |
| Third-generation cephalosporins | 0.1 | 4.8 | 6.17% | 0.007 | 8.2 | 10.59% | 1.72 |
| Other B-lactam antibacterials | 0 | 0.5 | 0.64% | 0 | 0.4 | 0.52% | 0.80 |
| Second-generation cephalosporins | 0.1 | 0.2 | 0.26% | 0.03 | 0.2 | 0.26% | 1.01 |
| Fluoroquinolones | 0.2 | 1.5 | 1.93% | 0.4 | 1.7 | 2.20% | 1.14 |
| Lincosamides | 0.2 | 0.4 | 0.51% | 0.3 | 1.4 | 1.81% | 3.52 |
| Tetracyclines | 0.7 | 1.1 | 1.41% | 0.02 | 0.8 | 1.03% | 0.73 |
| Carbapenems | 0 | 3.6 | 4.62% | 0 | 7.6 | 9.82% | 2.12 |

| | | | | | | | |
|---|---|---|---|---|---|---|---|
| Beta-lactamase resistant penicillins | 0.1 | 2.7 | 3.47% | 0.09 | 5.4 | 6.98% | 2.01 |
| Other antibacterials | 0.3 | 2.2 | 2.83% | 1.3 | 2.1 | 2.71% | 0.96 |
| Imidazole derivatives | 0.12 | 0.34 | 0.44% | 0.2 | 0.3 | 0.39% | 0.89 |
| Nitrofuran derivatives | 0 | 0 | 0.00% | 0.004 | 0.004 | 0.01% | NA |
| Glycopeptide antibacterials | 0 | 2.9 | 3.73% | 0 | 4.1 | 5.30% | 1.42 |
| Combinations of penicillins, incl. beta-lactamase inhibitors | 0.5 | 44 | 56.53% | 0.9 | 33.7 | 43.54% | 0.77 |
| Combinations of sulfonamides and trimethoprim, incl. derivatives | 0.2 | 0.8 | 1.03% | 0.4 | 1.5 | 1.94% | 1.89 |
| Polymyxins | 0 | 0.2 | 0.26% | 0 | 0.2 | 0.26% | 1.01 |
| Beta-lactamase sensitive penicillins | 0.01 | 0.7 | 0.90% | 0.06 | 0.9 | 1.16% | 1.29 |

DDD: daily defined dose; PD: patient day; RR: relative rate

**Table S4: Reported microbiological outcomes for the non-intervention and ASP MDT intervention periods**

| | *n* (rate)* | |
|---|---|---|
| | Non-intervention period | Intervention period |
| Rate of healthcare-associated Clostridium difficile infection in all adult patients | 0 | 6 (0.0008) |
| The 30-day all-cause readmission rate for patients with pneumonia | 17 (0.13) | 14 (0.2) |
| The 30-day all-cause readmission rate for patients with UTIs | 13 (0.16) | 21 (0.2) |
| | *n* (*n* per 100 patient days)** | |
| | Non-intervention | Intervention |
| MRSA bloodstream infection/100 adult patient days | 8 (0.00015) | 5 (0.0007) |
| Rate of healthcare-associated MDRO bloodstream infection/100 adult patient days. | 15 (0.0003) | 9 (0.00019) |

The number of cases is represented by *n*
**n* divided by the total number of cases (C.diff., UTI, or pneumonia)
***n* divided by total adult inpatient days then multiplied by 100.

**Table S5: Number of cultures growing ESBL producing bacteria**

| | **Non-intervention group** | **Intervention group** |
|---|---|---|
| **Community aquired** | 528 | 698 |
| **Hospital aquired** | 239 | 193 |
| **Total** | **767** | **891** |
